# Optical and Thermal Properties of Laser-Ablated Platinum Nanoparticles Graphene Oxide Composite

**DOI:** 10.3390/ijms20246153

**Published:** 2019-12-06

**Authors:** Amir Reza Sadrolhosseini, Mina Habibiasr, Suhaidi Shafie, Hassan Solaimani, Hong Ngee Lim

**Affiliations:** 1 Functional Device Laboratory (FDL), Institute of Advanced Technology, Universiti Putra Malaysia, Serdang, Selangor 43400UPM, Malaysia; suhaidi@upm.edu.my; 2 Department of Process and Food Engineering, Faculty of Engineering, Universiti Putra Malaysia, Serdang, Selangor 43400UPM, Malaysia; minahabibiasr@gmail.com; 3 Department of Fundamental and Applied Science, Universiti Teknologi PETRONAS, Seri Iskandar 3175, Malaysia; hassan.soleimani@utp.edu.my; 4 Department of Chemistry, Faculty of Science, Universiti Putra Malaysia, Serdang, Selangor 43400UPM, Malaysia; hongngee@upm.edu.my; 5 Material Synthesis and Characterization Laboratory (MSCL), Institute of Advanced Technology, Universiti Putra Malaysia, Serdang, Selangor 43400UPM, Malaysia

**Keywords:** Pt-NPs, graphene oxide, spatial self-phase modulation, laser ablation, platinum nanoparticles/graphene oxide composite, z-scan, thermal lens, photoacoustic

## Abstract

Platinum nanoparticles were synthesized in graphene oxide aqueous solution using a laser ablation technique to investigate the effect of optical linear, nonlinear and thermal properties of platinum-graphene oxide nanocomposite solution. The samples were prepared with different ablation times. The platinum nanoparticles that formed a spherical shape on the surface of graphene oxide solution were authenticated using UV-visible spectrum and transmission electron microscopy patterns. The particle size decreased with increasing ablation time, and the concentration and volume fraction of samples were increased. To obtain the optical linear, nonlinear and thermal properties of platinum-graphene oxide nanocomposite solution, UV-visible spectroscopy, Z-scan, thermal lens and photoacoustic techniques were used. Consequently, the linear and nonlinear refractive indices increased with an increase in the volume fraction of platinum nanoparticles. It was observed from the spatial self-phase modulation patterns that, the optical nonlinear property of the graphene oxide was enhanced in the presence of platinum nanoparticles, and the nonlinearity increased with an increase in the volume fraction of platinum nanoparticles inside the graphene oxide solution. The thermal diffusivity and thermal effusivity of platinum nanoparticles graphene oxide were measured using a thermal lens and photoacoustic methods, respectively. The thermal diffusivity and thermal effusivity of samples were in the range of 0.0341 × 10^−5^ m^2^/s to 0.1223 × 10^−5^ m^2^/s and 0.163 W s^1/2^ cm^−2^ K^−1^ to 0.3192 W s^1/2^ cm^−2^ K^−1^, respectively. Consequently, the platinum enhanced the optical and thermal properties of graphene oxide.

## 1. Introduction

In the field of nanotechnology, physical properties, as well as the use of metal nanoparticles, are interesting areas. The potentials of platinum nanoparticles (Pt-NPs) are great, as they can be widely applied for industrial and medical purposes. Pt-NPs have been employed in different fields which include the catalytic field [1], fuel cell [2], dye solar cell [3,4], sensor [5], remarkable catalytic in nano-medicine [6], anti-inflammatory [7], enzyme immobilization [8], electronic [9], automotive [10], and pharmaceutical industries [11]. Pt-NPs have a plasmonic property in the UV-visible range [12] and the heat generation efficiency of Pt-NPs is larger than that of other nanometals like Au-NPs [13]. Therefore, it is considered as the most appropriate candidate for hyperthermia therapy [14]. Pt-NPs have been synthesized in different shapes and sizes. One of the methods that can be used to efficiently prepare Pt-NPs is to reduce the chemical of platinum precursors in an aqueous solution. Usually, Pt-NPs are synthesized using a method that involves capping the Pt-NPs in any inorganic or organic solution. More so, the synthesis of Pt-NPs has been performed using iron liquid [15] surfactants [16], polymers [17,18,19], DNA [20], and proteins [21,22] as stabilizers. However, the use of such capping ligands partially blocks the pathways of electron-transfer or catalysis sites, thereby the catalytic activity of Pt-NPs is limited. In a study that was recently conducted, it was demonstrated that aggregation in nanoparticles can be prevented, while their catalytic activity is maintained when certain supports are used to anchor them [23]. Physical methods have been used to prepare the Pt-NPs, and the most dominant techniques used include sputtering and evaporation [12], ion implantation [24], ion implantation combine with surface etching, gamma radiolytic method [25], and laser ablation [26]. Laser ablation was used to disperse Pt-NPs in acetone [27], water [28], and methanol [26]. The effect of time and the pulsed energy were the significant parameters used for the evaluation of the shape and particle size of Pt-NPs [29]. Hence, Pt-NPs are capable of forming in organic and inorganic solutions using a laser ablation method. Therefore, the investigation of optical, thermal properties and formation of Pt-NPs in carbon-based nanomaterial solution is significant. 

Graphene oxide (GO) is a class of nanomaterials in a single layer form and it is formed by the oxidation of graphite. GO possesses some advantages, and one of them is solubility in an organic and inorganic solution like water and methanol. In this research, the GO was dissolved in water because the optical nonlinear and thermal properties of nanocomposite were desired. Carboxyl (–COO^−^) and hydroxyl (OH^−^) groups are the major functional groups of GO. More so, their interaction with other nanomaterial and molecular material like gold, silver, copper and zinc oxides is excellent [30]. Moreover, the composite of Pt-NPs and reduce graphene oxide was used to enhance the performance of a dye solar cell [31]. Hence, the investigation of Pt-NPs/GO nanocomposite aqueous solution is an important subject for some industrial applications.

The optical linear, nonlinear and thermal properties of nanomaterials are interesting subjects that can be used to evaluate nanomaterials and nanocomposites. The linear and nonlinear refractive indices and thermal properties of nanocomposites are the most significant parameters in nanophotonic. The UV-visible spectroscopy, Z-scan technique, spatial self-phase modulation (SSPM), thermal lens, and photoacoustic methods are some of the standard, versatile and commonly used methods of investigating the optical linear, nonlinear and thermal properties of nanomaterials. The refractive index and thermal properties of GO were reported in ref. [32], and the effects of nanomaterials such as gold and nickel were investigated. In this study, Pt-NPs were prepared inside the GO aqueous solution (Pt-NPs/GO) using the laser ablation methods. The prepared samples were characterized using UV-visible spectroscopy, Fourier transform infrared spectroscopy (FT-IR), and transmission electron microscopy (TEM). The real and imaginary parts of the refractive index and the absorption coefficient were derived from UV-vis spectrum. The nonlinear property of Pt-NPs/GO nanocomposite was investigated using Z-scan and SSPM pattern, whereas the thermal properties were investigated using thermal lens and photoacoustic methods to measure the thermal diffusivity, and thermal effusivity of prepared samples, respectively.

## 2. Results and Discussion

**Figure 1:**
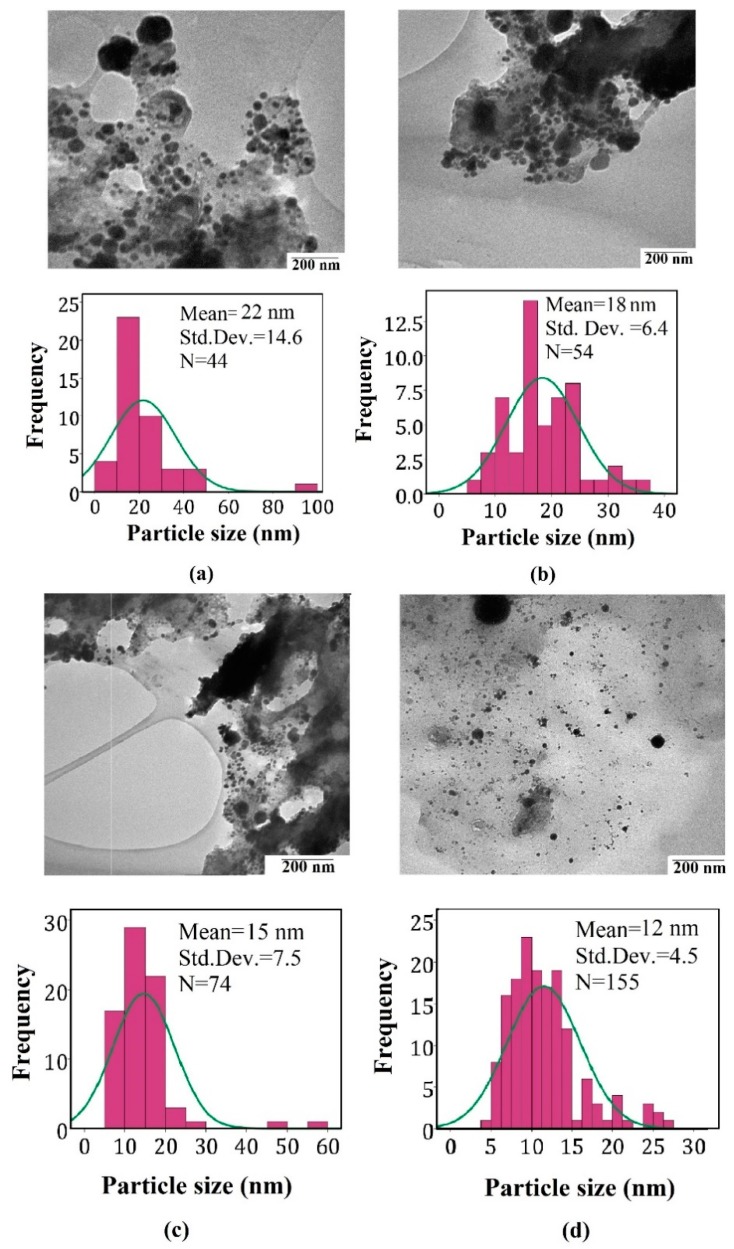
TEM images and analysis for samples (**a**) 5 min, (**b**) 10 min, (**c**) 20 min and (**d**) 30 min. The images confirm the formation of nanoparticles with a spherical shape.

Figure 1 shows the TEM image and analysis curve of Pt-NPs/GO at 5 min, 10 min, 20 min, and 30 min ablation times. The TEM images show the Pt-NPs formed in the solution contained the GO which surrounded the Pt-NPs and they were distributed in the solution. The images were analyzed using Image tools software ver.3. As a result, the Pt-NPs were distributed in the different sizes on the surface of GO. Histograms show the distribution of particle size, and the average particle size of Pt-NPs was found to be within the range of 22 nm, to 12 nm. The particle size decreased with an increase in the ablation time. The TEM images confirmed that the Pt-NPs formed a spherical shape, and were also distributed on the surface of the GO sheet.

**Figure 2:**
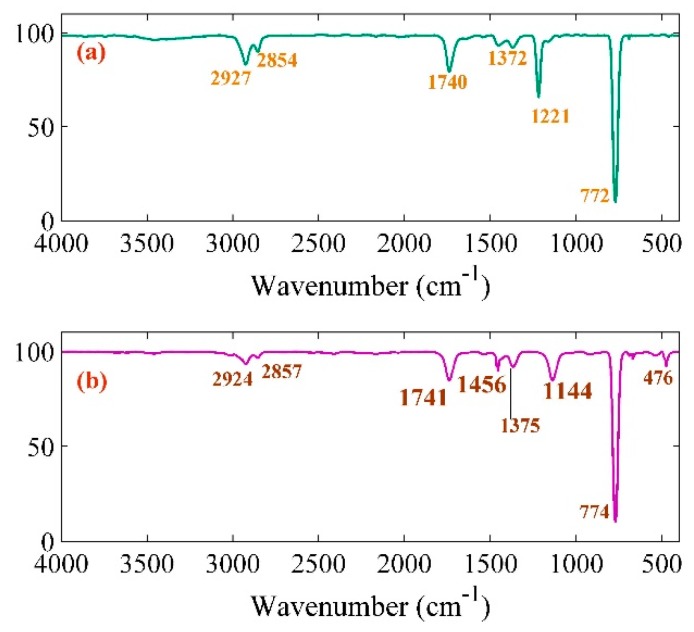
FT-IR result related to (**a**) pure GO and (**b**) Pt-NPs/GO composite.

Figure 2 represents the FT-IR spectrum of pure GO and Pt-NPs/GO composite ranging from 400 to 4000 cm^−1^. The main peaks were observed at 2927, 2854, 1740, 1372, 1221, 772, 2924, 2857, 1741, 1369, 1456, 1144, 774, and 476 cm^−1^. There was correspondence between the peaks at 2927, 2924, 2854, 2857 cm^−1^ symmetric and asymmetric stretching C–H bond in CH_2_ [33,34,35]. The C–O–C stretching, C–OH stretching, and aromatic C–H peaks were obtained at 1221, 1372, 1369, 722, and 774 cm^−1^, respectively. Moreover, peaks at 1456, 1144, and 476 cm^−1^ related to the OH deformation vibration [36] for C–O stretching, the COOH vibration bands, and the Pt-NPs, respectively. Therefore, the observation of two new peaks at 1456 and 1144 cm^−1^ is an indication that there was a strong connection between COO– and OH to the Pt-NPs [37,38]. Therefore, there was strong capping of the Pt-NPs by functional groups of GO plane like the COO– and OH group [39].

**Figure 3:**
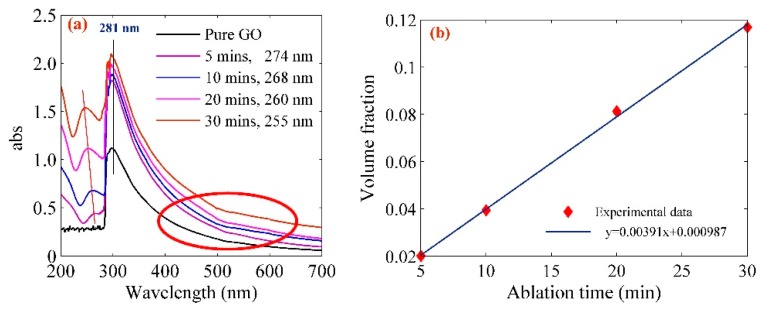
(**a**) UV-vis spectrum of Pt-NPs/GO composite shows the peaks at 281 nm, 274 nm, 268 nm, 260 nm, and 255 nm which confirm the blue shift and formation of Pt-NPs in GO. The red ellipse area shows the absorbance increased as the ablation time increased, (**b**) variation of volume fraction with ablation time.

It can be observed from Figure 3a that the UV-visible spectrum of the Pt-NPs/GO composite is within the range of 200 nm and 700 nm. The spectrum of pure GO and Pt-NPs/GO shows a peak ranging from 281 nm to 255 nm referring to the n-π* of the C=O bond transition and Pt (*n* = 6, *l* = 0) absorption. A blue shift was observed in the spectrum of Pt-NP/GO, which according to Mie theory, is attributed to a decrease in the particle size [40,41,42]. The measurement of the Pt-NPs/GO concentration was done using atomic absorption spectroscopy which increased from 4.3 to 25 ppm. The volume fraction of the Pt-NPs which was dispersed in GO solution was calculated from the volume of the Go solution (*V_L_*) and the volume of Pt-NPs as follows [43]:

(1) $$V=\frac{V_{p}}{V_{p}+V_{L}}=\frac{C_{\text{Pt-NPs}}}{C_{\text{Pt-NPs}}+\rho }$$

where *V*_L_ and *V*_p_ are the volume of the GO solution and the volume of Pt-NPs, respectively. *V*_p_ is the ratio of mass and density (*m*/*ρ*) of Pt-NPs [44]. The pertinent parameters are listed in Table 1.

**Figure 4:**
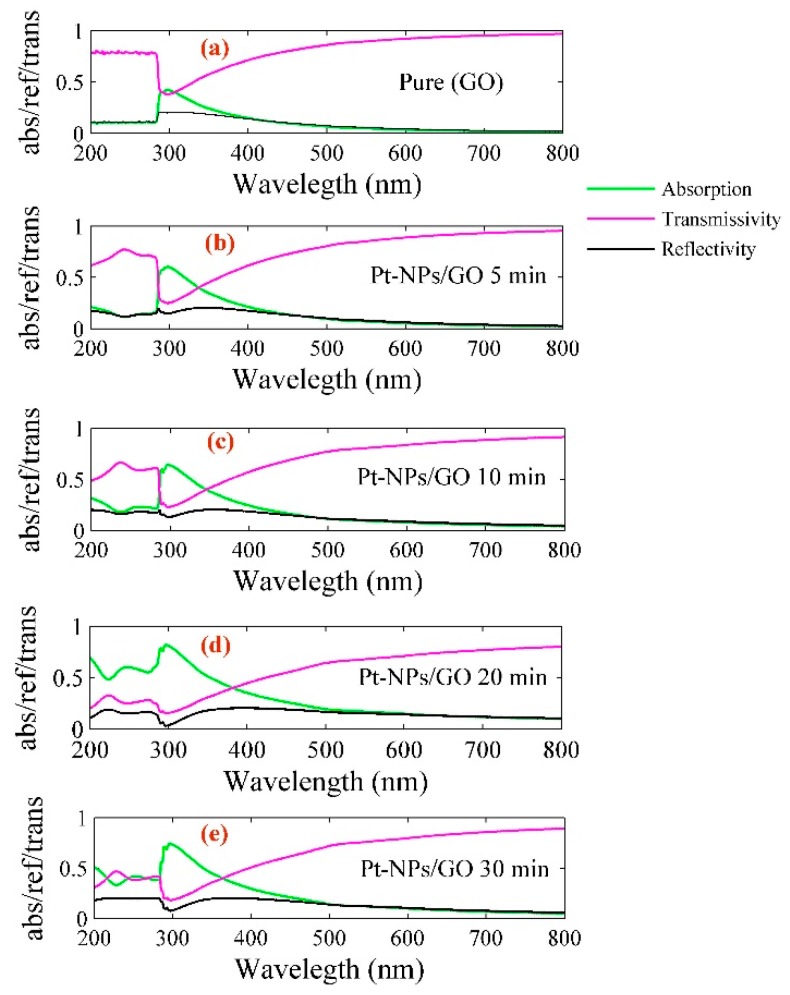
Variation of absorption, transmissivity, and reflectivity of (**a**) pure GO and (**b**) Pt-NPs/GO at 5 min, (**c**) Pt-NPs/GO at 10 min, (**d**) Pt-NPs/GO at 20 min, (**e**) Pt-NPs/GO at 30 min nanocomposite with wavelength.

Pt-NPs were formed in the GO solution with different sizes. The optical properties of Pt-NPs/GO composite are a significant and interesting subject in this study. Figure 4 shows the reflectivity and transmissivity of Pt-NPs/GO nanocomposite, with the spectra ranging from 200 nm to 80 nm. Based on Beer Lambert’s law, the real and imaginary pars of refractive index and absorption coefficient of Pt-NPs/GO can be derived from reflectivity and transmissivity spectra. The extinction and absorption coefficients of Pt-NPs/GO composite can be used as scales for the evaluation of the optical properties of nanocomposites such as absorption and transmission.

**Figure 5:**
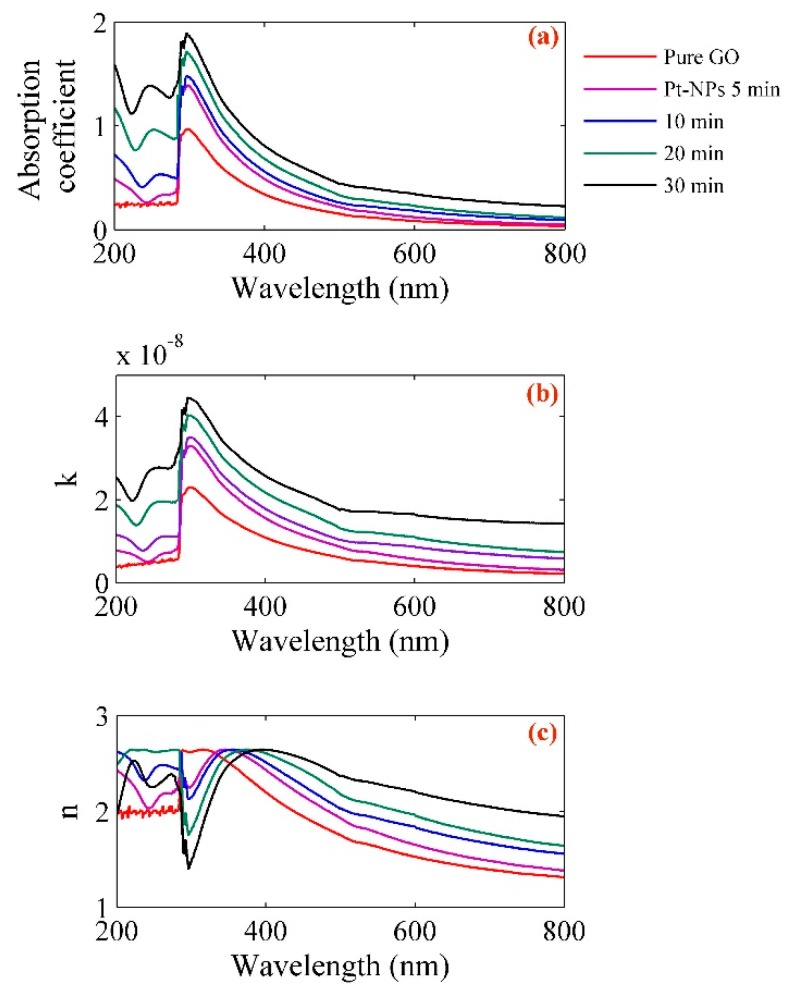
Variation of (**a**) absorption coefficient, (**b**) imaginary and (**c**) real parts of refractive index for pure GO, Pt-NPs/GO composite with wavelength in different ablation time.

Figure 5 presents the analysis of optical parameters. The coefficients of absorption, imaginary parts and real parts of refractive index for different concentration of Pt-NPs/GO composite were calculated based on Figure 4. The absorbance and optical density of the Pt-NPs/GO composite are proportional to transmissivity (*T*) as follows [43]:

(2) $$absorbance=optical_{{}_{{}_{}}}density=-\log_{10}^{}T=-\log_{10}^{}(\frac{I}{I_{0}})$$

where *I*_0_ and *I* denote the intensity of the incident light beam and the intensity of the transmitted beam, respectively. The calculation of the optical density of the Pt-NPs/GO composite can be done from the optical path (*l*) and the absorption coefficient (*α*) as follows:

(3) OD=0.434αl

In this experiment, the optical path was 10 mm, and the absorption coefficients of Pt-NPs/GO for 5, 10, 20, and 30 min were obtained as 0.17635, 0.22797, 0.28831 and 0.0541, respectively. Moreover, the real (*n*) and imaginary parts of the refractive index of Pt-NPs/GO are a function of the wavelength (λ) and absorption coefficient (*α*) as follows [45]:

(4) $$k=\frac{\alpha \times \lambda }{4\pi }$$

(5) $$R=\frac{{\left(n-1\right)}^{2}+k^{2}}{{\left(n+1\right)}^{2}+k^{2}}$$

where *R* is reflectivity.

The important values of the refractive index of Pt-NPs/GO for 532 nm, 594 nm, and 633 nm were derived from Figure 5, and they have been sorted in Table 2.

**Figure 6:**
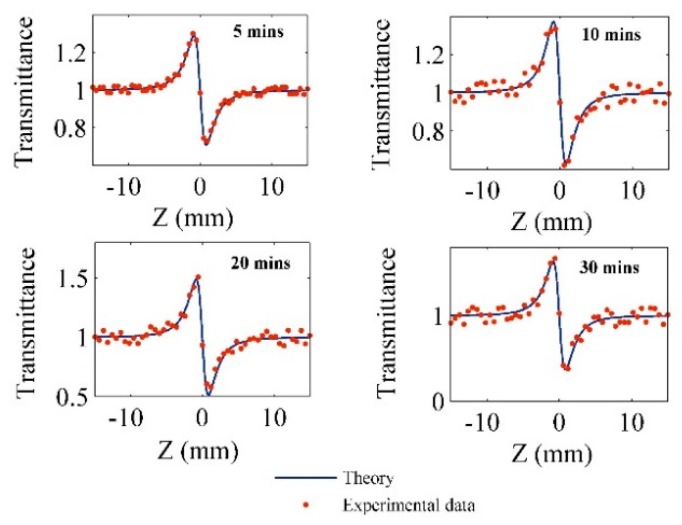
The Z-scan results show the variation of transmittance of the laser beam with distance for different ablation times of Pt-NPs in GO solution.

In order to obtain the nonlinear refractive index, the Pt-NPs/GO solutions were tested using a homemade Z-scan setup (see Figure 12). Figure 6 shows the Z-scan signals of the Pt-NPs/GO composite solution for different ablation times. Variation of laser beam intensity (transmittance ΔT) with distance was considered in the range of −15 mm to 15 mm around the focal point. The transmittance is proportional to the phase shift and Rayleigh length ($z_{0}$) as follows [46]:

(6) $$\Delta T(z)=1-\frac{4\Delta \varphi_{0}x}{\left(x^{2}+1)(x^{2}+9\right)}$$

where $\Delta \varphi_{0}$ and x are the phase change and normalized distance, respectively. The normalized distance ($x=z/z_{0}$) is proportional to the Rayleigh length ($z_{0}$) and the movement distance of sample (*z*) [47].

In Figure 6, the dotted points are the experimental values of transmittance, which are well fitted into the theoretical formula Equation (6) for the purpose of obtaining the phase shift within the range of −1.43 to −3.1. Moreover, the interaction length of the laser beam (sample thickness) with the samples was 1 mm, and the effective lengths of nonlinear medium were calculated as follows:

(7) $$L_{eff}=(1-\exp(-\alpha_{0}L))/\alpha_{0}$$

where $\alpha_{0}$ and L are the linear absorption coefficient of the samples that were derived from Figure 5, and the sample thickness, respectively. Therefore, the effective nonlinear length is within the range of 9.9991 to 9.9979, and it decreased as the ablation time increased.

In accordance with existing literature, the nonlinear refractive index ($n_{2}$) of the Pt-NPs/GO solution corresponds to phase change as follows [36]:

(8) n2=Δφ0kLeffI0

where $I_{0}$ and k=2π/λ are the on-axis irradiance at focal point and the wave vector, respectively.

Therefore, the phase shift and effective nonlinear length were achieved using equations (6) and (7). Then, the nonlinear refractive indices of Pt-NPs/GO were calculated from Equation (8) [39]. Consequently, the nonlinear refractive index fell within the range of −1.56 × 10^−9^ to −3.81 × 10^−9^. The pertinent parameters are sorted and presented in Table 3.

**Figure 7:**
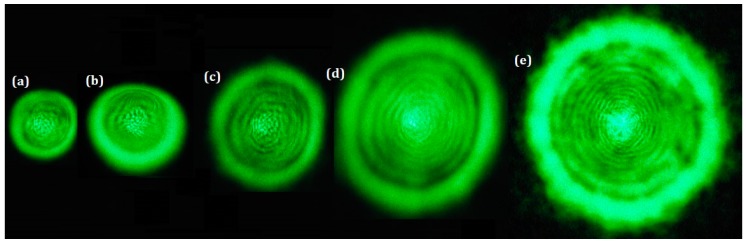
The SSPM pattern for (**a**) pure GO, Pt-NPs/GO (**b**) 5 min, (**c**) 10 min, (**d**) 20 min, (**e**) 30 min ablation time.

The SSPM setup (Figure 13) was used to investigate the SSPM properties of the Pt-NPs/GO solution. Figure 7 depicts the spatial self-phase modulation (SSPM) pattern for pure GO and Pt-NPs/GO composite in 5 min, 10 min, 20 min, and 30 min. Through the use of solid-state laser at 532 nm wavelength, the experiments were performed. The emergence of the SSPM patterns in the far field occurs as a result of the passage of the green intense Gaussian laser beam through the samples. After the absorption of the laser beam by the Pt-NPs/GO, the appearance of the nonlinearity properties of Pt-NPs/GO was noticed, and this appearance results from the change in the refractive index of Pt-NPs/GO solution. Thus, the interaction that occurred between intense laser beam and nanocomposite caused the change in the phase of the laser beam nanocomposite because of the variation in the optical paths in the medium as a nonlinearity of Pt-NP/GO.

The primary absorption peak of GO is around 280 nm, while within the green range it has no considerable peak absorption. Figure 3a shows the absorbance increased when the ablation time shifted from 5 min to 30 min (red ellipse) and Figure 5a shows the absorption coefficient of nano composites in the 532 nm is in the range of 0.2314 to 0.4496. Thus, the photon energy of laser beam can be absorbed by the Pt-NPs/GO. More so, there was scattering of laser beams, and then there was interference of the beams in the field. Based on the result produced by TEM, a decrease occurred in the size of the particle as the ablation time increased. With a decrease in the size of particle, an increase occurred in the scattering of laser beam, while the strength of the SSPM increased [48]. According to the Equation (2), the intensity of SSPM pattern is a function of wavelength and the absorption coefficient. Therefore, as an increase occurred in the concentration of Pt-NPs and ablation time, the number of diffraction rings and radius of diffraction rings, as well as the pattern size increased.

**Figure 8:**
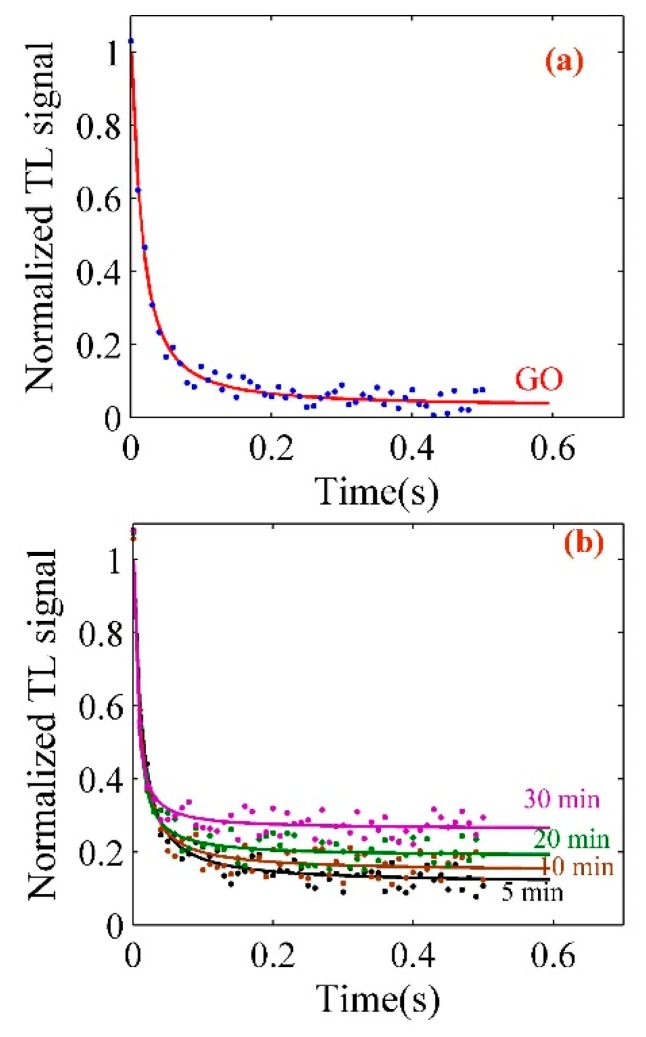
(**a**) Thermal lens signals related to GO solution; (**b**) thermal lens signals to determine the thermal diffusivity of the Pt-NPs/GO solution at different ablation times.

The thermal diffusivity of pure GO, Pt-NPs/GO composite solutions was measured using a double bean thermal lens technique (Figure 14). The concentration Pt-NPs/GO composite was in the range of 4.3 ppm to 25 ppm, and the experiments were carried out at room temperature with He-Ne laser and high-power green laser. Figure 8a,b depict the TL signals for GO and Pt-NPs/GO composite solutions at different concentrations. The signals were detected using a photo-diode. GO or Pt-NPs/GO was a probe medium and they scattered the He-Ne laser. The intensity of the He-Ne laser beam was a function of time. The TL phenomenon depends on the absorption of high power laser energy with GO or Pt-NPs/GO and heat was generated in the medium. Therefore, there was an interaction between the excitation laser beam (high power laser) and the GO or Pt-NPs/GO, thereby leading to an increase in the local temperature.

The thermal lens signal is proportional to the thermal time constant ($t_{c}$), and refractive index gradient (θ) with temperature. Hence, the experimental data were analyzed using Equation (21), and the main parameters such as θ and $t_{c}$ were achieved by Equation (9) as a minimum root square algorithm [49].

(9) $$\Gamma ={\sum \left(I_{Theory}(t)-I_{Exper}(t)\right)}^{2}$$

where the theoretical and the experimental intensity of the probe laser beam is depicted as $I_{Theory}$ and $I_{Exper}$, respectively. The thermal diffusivity (D) was obtained using Equation (10) by substituting the values of $\omega_{e}$ and $t_{c}$, which were derived from the analysis of thermal lens signals (Figure 8a,b). The parameters are listed in Table 4. The validity of the thermal diffusivity of GO and Pt-NPs/GO was calculated as follows [50]:

(10) $$\Delta D=2D\cdot (\frac{1}{\omega_{e}}\Delta \omega_{e}+\frac{1}{2t_{c}}\Delta t_{c})$$

where $\Delta \omega_{e}$ and $\Delta t_{c}$ were 0.0001 and 0.000002, respectively. Hence, the sources of error are the limitations of thermal time constant measurement, whereas the excitation beam radius is the main source of measurement limit and the average accuracy is 0.65 × 10^−6^ m^2^/s. As a result, the thermal diffusivity of Pt-NPs/GO increased as the particle size decreased, thereby increasing the volume fraction. This is because the scattering cross section of the light beam increased.

**Figure 9:**
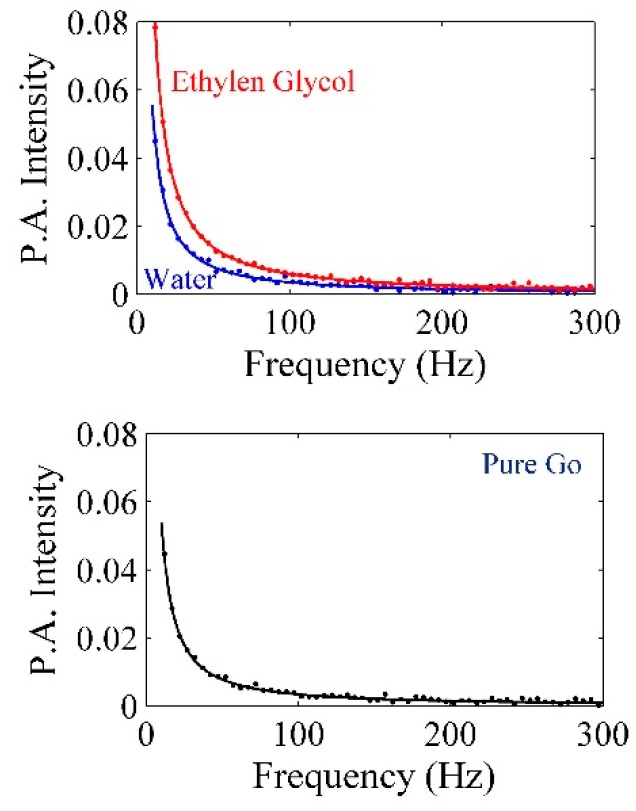
PA signal for calibration of set up in the presence of deionized distilled water and ethylene glycol to find the $P_{1}$ and $P_{2}$.

**Figure 10:**
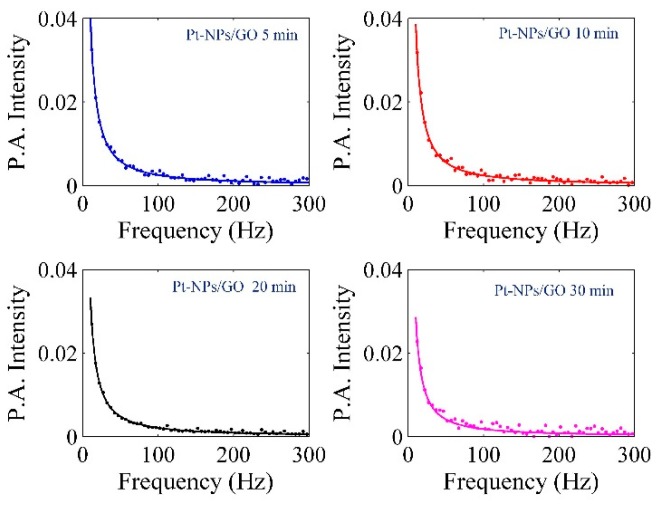
PA signals related to the measurement of thermal effusivity of GO and Pt-NPs/GO solution for 5, 10, 20, and 30 min ablation times.

Transfer and exchange of heat can be achieved by thermal effusivity (ε) in the presence of the given nanomaterial, and thermal effusivity has an association with the thermal impedance of Pt-NPs/GO. The PA signals were achieved using a homemade PA setup (Figure 16). The PA signals of water ethylene glycol, pure GO, and Pt-NPs/GO are presented in Figure 9 and Figure 10. The PA signals were registered for deionized distilled water, ethylene glycol and different concentration of Pt-NPs/GO composite solutions. The first step involves calibrating the setup through the use of ethylene glycol and deionized distilled water, ethylene glycol, and the constant parameters were obtained. The thermal effusivity was obtained using the Rosencwaig Gersho (RG) with the aim of analyzing the signals [32]. Thus, the amplitude of the PA signals is inversely proportional to the frequency of chopper as a modulation frequency [51] of the laser beam, and the calculation of the amplitude of pressure fluctuations was done in the presence of the solution as follows [52]:

(11) |δP|=P1fP2(1+P3f+P322f)12

where

(12) P3=2εsεAllAl(αAlπ)12

$l_{Al}$, $\varepsilon_{Al}$, and $\alpha_{Al}$ represent the thickness of aluminum foil, thermal effusivity, and the thermal diffusivity, respectively. $P_{1}$ and $P_{2}$ have been achieved from the Al foil signal. They are proportional to chopper frequency (modulation frequency). $P_{1}$ and $P_{2}$ are adjustable parameters that should be obtained by fitting the experimental data to Equation (13) [52]:

(13) $$\left|\delta P_{Al}\right|=P_{1}f^{P_{2}}$$

The thermal diffusivity and the thickness of aluminum were constant, and they were equivalent to 0.0017, 0.99 mm. Consequently, $P_{1}$, $P_{2}$, and $\varepsilon_{Al}$ have been achieved from Figure 9 as 34.7867, 1.456, and 2.36, respectively. After that, the GO, Pt-NPs/ GO composite solutions were separately loaded to the sample tank, and the PA signals that were registered are shown in Figure 10. The results have been presented in Table 4. As a result, when the ablation time increased, the particle size decreased while the volume fraction increased. This indicates that the scattering cross section of phonons increased. More so, as the concentration of Pt-NPs increased, the thermal effusivity increased.

## 3. Materials and Methods

### 3.1. Laser Ablation 

**Figure 11:**
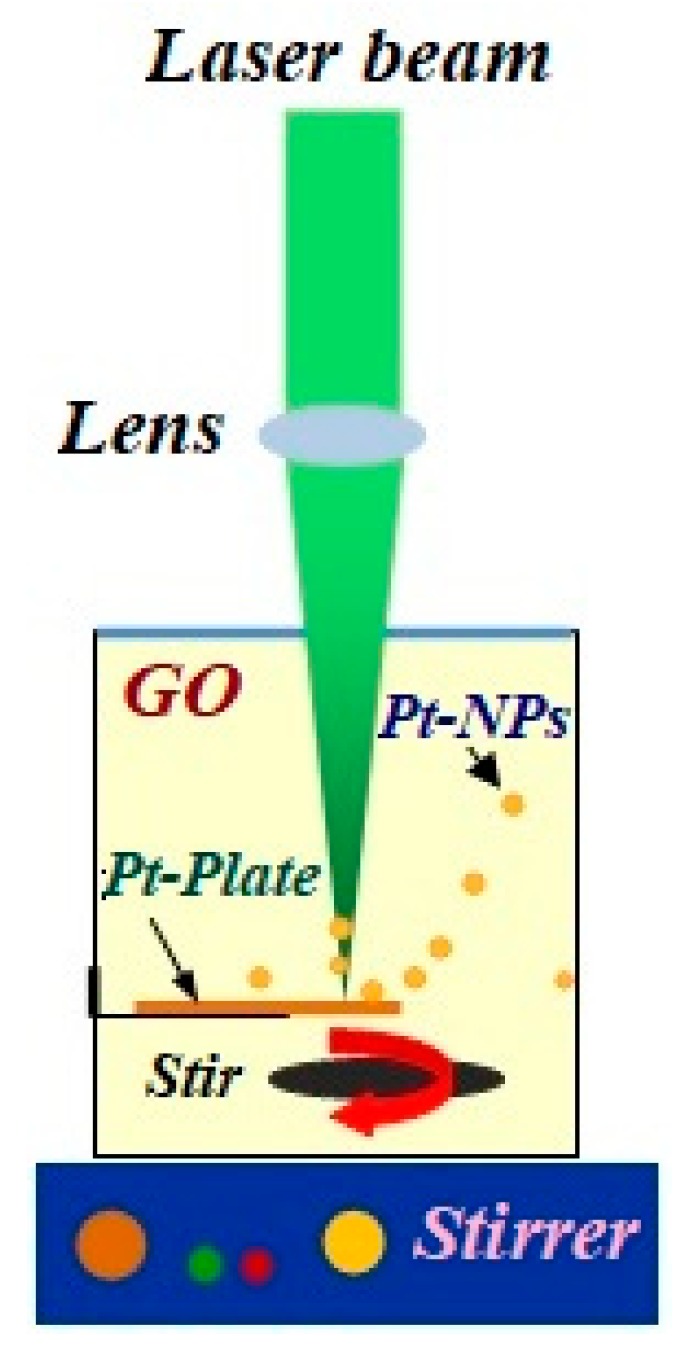
Laser ablation setup contains Nd:YAG, lens, stirrer and pure Pt plate.

The Hummer’s method which was reported by Huang et al. 2011 [53], was used in producing the grapheme oxide alongside the graphite oxidation. The GO was dissolved in water and the concentration of GO which was used in this experiment was 0.1 mg/mL. The platinum plate (Aldrich, 99.99%) was immersed in 15 mL of GO solution. The laser ablation method is demonstrated in Figure 11 with the setup containing a lens, a high power laser, a stirrer and a liquid cell [54,55,56]. The Pt plate was ablated using an Nd:YAG Q-Switch pulsed laser beam. The wavelength, energy and reputation rate of the laser beam were 532 nm, 1200 mJ, and 40 Hz, respectively. The nanoparticles were dispersed in the GO solution using the stirrer while the Pt plate was ablated. The time used in the ablation was 5, 10, 20, and 30 min. A UV-visible spectrometer (Perkin Elmer, Parstat 2263, Boston, MA, USA) and transmission electron microscopy (TEM, HitachiH-7100, Tokyo, Japan) were employed in obtaining the morphology and particle size of Pt-NPs.

### 3.2. Z-Scan Setup

**Figure 12:**
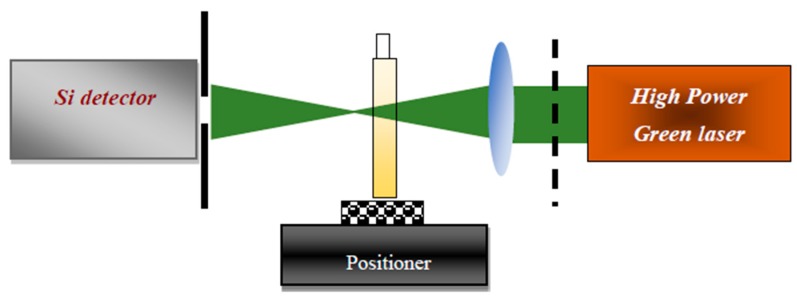
The Z-scan setup containing high power green laser, lens, chopper, tank, pinhole, and detector.

Figure 12 shows the homemade Z-scan setup containing a solid-state laser (532 nm), a lens, a pinhole, a chopper, a silicon detector, and a Lock-in amplifier. The sample holder was placed on the optical bench which was connected to a precision traveling stage. The sample was moved from −15 mm before the focal point of the lens to +15 mm after the focal point of the lens. The Z-scan signal was registered when the precision traveling stage stopped for a while [39,57]. 

### 3.3. Spatial Self-Phase Modulation Setup

**Figure 13:**
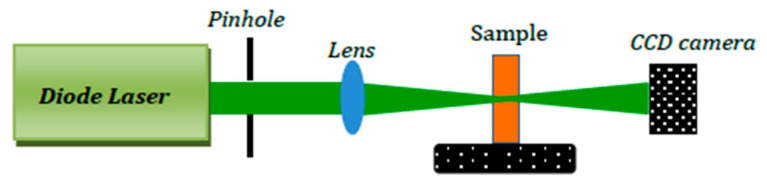
The SSPM setup contains a high power diode laser, pinhole, quartz cuvette, and CCD camera.

The use of spatial self-phase modulation (SSPM) was employed in investigating the nonlinear optical property of Pt-NPs/GO. The SSPM setup is presented in Figure 13. The setup consists of a green laser with a 532 nm wavelength and 150 mW power, a pin hole, a quartz cuvette cell (*L* = 2 mm) and a lens (*f* = 30 mm). The testing of the samples was done separately, and the CCD camera was used for registering the SSPM patterns when the samples were located at the divergence point of the incident high power laser beam after the focal point of lens [58]. The CCD camera was positioned 50 cm behind the exit plan of the quartz cuvette. 

The nonlinear properties possessed by organic and nanomaterial solution, are referred to as Spatial self-phase modulation (SSPM). This study involved the investigation of the SSPM for GO and Pt-NPs/GO solutions through the use of green (532 nm) laser. SSPM is related to the variation of refractive index in if high intensity passes through the medium. The difference in refractive index on the far field diffraction pattern causes the phenomenon to depend on a phase shift. The analysis of the SSPM patterns was done using a Fraunhofer’s approximation of the Fresnel-Kirchhoff diffraction equation as follows [59]:

(14) $$I=I_{0}{\left|\int_{0}^{\infty }J_{0}(k_{0}\theta r)\exp\left[-\frac{r^{2}}{\omega_{p}^{2}}-i\phi (r)\right]rdr\right|}^{2}\omega_{p}$$

where θ, ϕ, $\omega_{p}$, r and $k_{0}$ are the far field diffraction angle, phase on exit plane, beam radius at the medium, radial coordinate and wave number in free space, respectively. $I_{0}$ is the proportion of the linear absorption coefficient (α), wavelength (λ), thickness of medium along the propagation of beam (L) and distance from the medium to the observational plane (*D*).

(15) $$I_{0}=4\pi^{2}{\left|\frac{E(0,z_{0})}{i\lambda D}\exp(\frac{-\alpha L}{2})\right|}^{2}$$

When a Gaussian laser beam propagates through the nonlinear medium, the transverse phase shift on the exit Gaussian laser beam is expressed as follows [60,61]:

(16) $$\Delta \phi (r)=k_{0}\int_{z_{0}}^{z_{0}+L}\Delta n(z,r_{1})dz$$

(17) $$\Delta n(z,r_{1})=n_{2}I(z,r_{1})$$

where $\Delta n(z,r_{1})$ is denoted as the refractive index distribution, and is proportionate to the laser beam intensity and nonlinear refractive index ($n_{2}$). The complex light field amplitude on the exit plane of the nonlinear medium is considered as follows:

(18) $$E(r_{1},z_{0}+L)=E(0,z_{0})\exp(-\frac{\alpha L}{2})\exp(-\frac{r_{1}^{2}}{\omega_{p}^{2}})\exp(-i\phi (r_{1}))$$

where α and L are the linear absorption coefficient and nonlinear medium length, respectively, and the total phase shift due to the induced transverse additional phase shift and the Gaussian phase shift that originates is as follows:

(19) $$\phi (r_{1})=k_{0}\frac{n_{0}r_{1}^{2}}{2R}+\Delta \phi (r_{1})\approx k_{0}\frac{n_{0}r_{1}^{2}}{2R}+\Delta \phi_{0}(z_{0})\exp(-\frac{2r_{1}^{2}}{\omega_{p}^{2}})$$

$\Delta \phi_{0}$ is the peak of the nonlinear phase shift induced in the beam and is proportional to nonlinear refractive index of the medium as [61]

(20) $$\Delta \phi_{0}=k_{0}\Delta n(z_{0},0)L$$

### 3.4. Thermal Lens Setup

**Figure 14:**
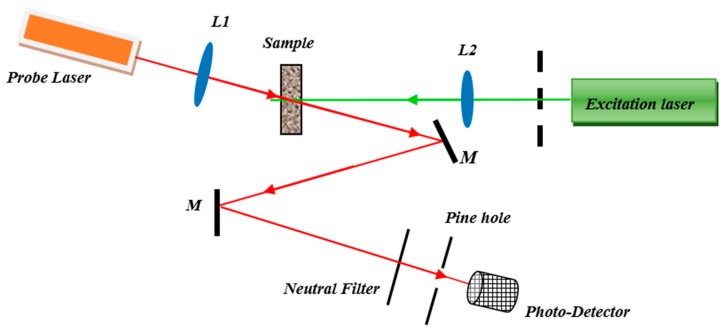
The homemade thermal lens double beam setup for the measurement of thermal diffusivity of Pt-NPs/GO composite.

The double beam thermal lens setup was used to measure the thermal diffusivity of Pt-NPs/GO composite. The set up (Figure 14) contains a He-Ne laser ( 632.8 nm and 0.5 mW, model: R-31003, Newport, Irvine, CA, USA), a high-power solid-state laser (532 nm at 80 mW, model: LRS-0532, Laserglow Technology, Toronto, Canada) two lenses, a beam splitter, a silicon detector (model: 818-SL, Newport, Irvine, CA, USA), chopper (Stanford research, RS540, Sunnyvale, CA, USA) and the Lock-in amplifier [49]. The solid-state laser and He-Ne laser were used as an excitation light and as a probing beam, respectively. Figure 14 shows the thermal lens setup, where the sample is located at the focal point of lens (*L1*, *f* = 21 cm) and the solid-state laser beam was focused onto the Pt-NPs/GO composite solution. The chopper modulated the He-Ne laser beam at 10 *Hz* frequency, and it focused on the sample using a second lens (*L2*) with shorter focal length (*f =* 10 cm) than the first lens (*L1*). The angle between the excitation beam and the probe beam was less than 10°. An optical neutral filter and pinhole were used to control the intensity and the path of solid-state laser beam from the excitation of the sample to enter the silicon detector. The thermal lens signal was registered using the silicon detector when it was coupled with the Lock in amplifier [62,63].

**Figure 15:**
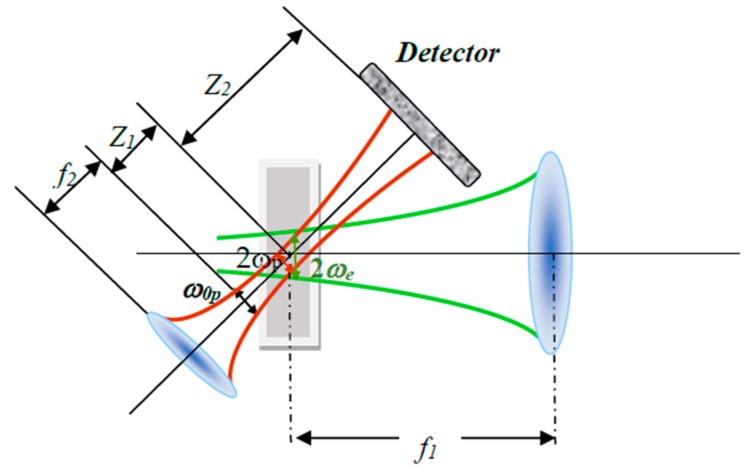
The mechanism and parameters of thermal lens.

Figure 15 shows the mechanism and parameters of thermal lens (TL). The TL phenomenon appeared when the probe medium such as, GO or Pt-NPs/ GO composite solution absorbed high power laser energy and heat was generated in the medium.

After that, the probe medium scatters the He-Ne laser and the intensity of laser beam was a function of time. Therefore, the excitation laser beam (high power laser) and the medium interact together; thereby, the local temperature increases. Therefore, the thermal lens was generated like a normal optical lens. Consequently, when the He-Ne beam passed through the medium, the light beam focused or scattered, and the photo-detector was able to register the variation of light intensity of He-Ne laser with time.

The TL signals were analyzed using thermal lens spectroscopy theory, which was investigated by Shen at. al in 1992 [50]. They explained the variation of probe laser intensity with time, and presented the intensity formula as follows [50]:

(21) $$I\left(t\right)=I\left(0\right){\left[1-\frac{\theta }{2}arctan\left(\frac{2mV}{{\left(1+2mV\right)}^{2}\frac{t_{c}}{2t}+1+2m+V^{2}}\right)\right]}^{2}$$

where θ and $t_{c}$ are thermal time constant and refractive index gradient with temperature, respectively.

(22) $$t_{c}=\frac{\omega_{e}^{2}}{4D}$$

and *I* (0) (*t* = 0) is the initial intensity. *D* and *ω_e_* are the thermal diffusivity and the excitation beam radius at the probe medium. Consequently, thermal diffusivity is a function of time and thermal time constant. Moreover, θ depends on the parameters of high-power laser and change of refractive index of probe medium with the temperature of the sample as follows:

(23) $$\theta =\frac{p_{e}A_{e}L}{\kappa \lambda_{p}}\left(\frac{dn}{dT}\right)$$

where $p_{e}$, $A_{e}$, L, $\lambda_{p}$, T and κ are the excitation beam power, the absorption coefficient, the sample thickness (10 mm), the probe beam wavelength, temperature and the thermal conductivity of the sample [43,44], respectively. $\frac{dn}{dT}$ is variation of the refractive index in the probe medium. V, m are parameters related to beam radius and place of probe medium.

(24) $$V=\frac{Z_{1}}{Z_{c}};\;Z_{C}=\frac{\pi \omega_{0p}^{2}}{\lambda_{p}}$$

(25) $$m={\left(\frac{\omega_{p}}{\omega_{e}}\right)}^{2}$$

The $\omega_{0}$, $\omega_{p}$,$\omega_{e}$, $Z_{1}$, and $Z_{c}$ are the probe beam waist radius, the probe beam radius at sample, the excitation beam radius at the sample, the distance of the laser beam waist from the sample, and the confocal distance for the probe laser beam (L2; $Z_{c}$= *f =* 0.12 m) at wavelength (*λ_p_*) [50,64], respectively. Normally, the thermal diffusivity can be obtained from thermal time constant that can be derived from analysis the TL signal.

Figure 15 [50] shows thermal lens parameters. $\omega_{p}$, $\omega_{e}$, *f*_2_ and *Z*_2_ are probe beam radius, the excitation beam radius at solutions, second focal distance and the distance between solution and the laser beam waist, respectively [64]. The solution and photodetector were placed at $\sqrt{3}Z_{c}$ and *Z*_2_ from solution.

### 3.5. Photoacoustic (PA) Setup 

**Figure 16:**
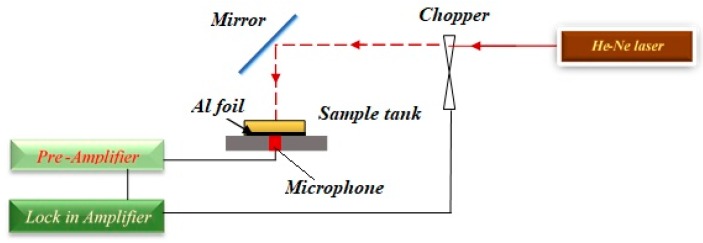
Photoacoustic setup containing He-Ne laser, chopper, mirror, sample tank, Al foil, microphone, pre amplifier, and lock in amplifier.

The PA setup containing a microphone, a plan mirror, lock-in amplifier, a sample tank, He-Ne laser (75 *mW*, 632.8 nm), a chopper, and a pre-amplifier, is shown in Figure 16. The use of a chopper was employed in modulating the laser beam, and the frequency of chopper moved from 21 *Hz* to 236 *Hz*. The sample tank’s bottom was covered using aluminium foil with a thickness of 0.017 mm. Contact between the Pt-NPs/GO composite solution and aluminium foil was established. The microphone which was connected to a pre-amplifier and lock-in amplifier was used in registering the photoacoustic signals. The amplitude and phase the PA signals were a function of a light beam chopper frequency. The measurement of the thermal effusivity of Pt-NPs/GO was performed at room temperature. The empty sample tank was measured repeatedly so as to measure the thermal effusivity. For the analysis of the phase and amplitude of the PA signal, the Rosencwaig–Gersho (RG) theory was used with the aim of obtaining the thermal effusivity of Pt-NPs/GO composite solution [32,65].

## 4. Conclusions

Pt-NPs were formed in the GO solution using laser ablation technique. The UV-visible spectrum, TEM images, and FT-IR spectrum confirmed the formation of spherical-shaped Pt-NPs in the GO solution, and the interaction between Pt-NPs/GO and carboxylic and hydroxylic functional group at the edge and surface of GO. The concentration of nanoparticle increased when the laser ablation time was increased. The particle size was in the range of 12 nm to 22 nm, and it decreased with an increase in the ablation time, while the volume fraction also increased. The linear, nonlinear refractive indices, thermal diffusivity and thermal effusivity of Pt-NPs/GO composite solution increased as an increase occurred in the volume fraction of composite. The SSPM pattern appeared in the far field, and an increase was observed in the size of pattern, radius of diffraction rings, and number of diffraction rings as an increase occurred in the volume fraction and concentration of Pt-NPs.

## Figures and Tables

**Table 1:** Concentration and particle size of Pt-NPs in GO solution.

Pt-NPs/GO (min)	Concentration of Pt-NPs (ppm)	Particle Size (nm)	Volume Fraction of Pt-NPs
5	4.3	22	0.02009
10	8.43	18	0.03939
20	17.4	15	0.08131
30	25	12	0.11683

**Table 2:** The real and imaginary parts of the refractive index of Pt-NPs/GO composite at different ablation times for 532 nm, 594 nm and 633 nm wavelengths.

Sample	*n*			k (10^−8^)		
	532 (nm)	594 (nm)	633 (nm)	532 (nm)	594 (nm)	633 (nm)
Pt-NPs (5 min)	1.82334	1.80125	1.58539	7.466	7.251	0.499
Pt-NPs (10 min)	1.95732	1.94191	1.76538	9.636	9.544	0.785
Pt-NPs (20 min)	2.09519	2.07813	1.87933	12.206	12.071	0.993
Pt-NPs (30 min)	2.31368	2.30018	2.14037	17.151	17.096	1.562

**Table 3:** The phase shift, linear absorption coefficient, effective nonlinear length and nonlinear refractive index.

Pt-NPs/GO (min)	$\Delta \varphi_{0}$	$\alpha_{0}$	$L_{eff}$	$n_{2}(10^{-9})$
5	−1.435	0.176	9.9991	−1.56
10	−1.83	0.228	9.9988	−1.79
20	−2.4	0.288	9.9985	−2.43
30	−3.1	0.405	9.9979	−3.81

**Table 4:** Measurement parameters indicating the thermal properties of the solution.

Sample	$P_{3}$	Thermal Effusivity (W s^1/2^ cm^−2^ K^−1^)	θ	$t_{c}\;$	Thermal Diffusivity (10^−5^ m^2^/s)	$\Delta D\;(10^{-7})$
Water	45.614	0.163			-	
Ethylene glycol	26.025	0.093			-	
Pure GO	47.013	0.168	1.78	0.00319	0.0341	0.0763
Pt-NPs/GO 5 min	63.076	0.2254	1.48	0.00308	0.0432	0.096
Pt-NPs/GO 10 min	65.818	0.2352	1.39	0.00227	0.0585	0.132
Pt-NPs/GO 20 min	76.396	0.273	1.30	0.00152	0.0875	0.202
Pt-NPs/GO 30 min	89.325	0.3192	1.15	0.0011	0.1223	0.288

## Abbreviations

Pt-NPs	Platinum nanoparticles
GO	Graphene oxide
SSPM	Spatial self-phase modulation
PA	Photoacoustic
RG	Rosencwaig Gersho
TEM	Transmission electron microscopy
TL	Thermal lens
FT-IR	Fourier transform infrared spectroscopy

## References

[1] Duan S., Du Z., Fan H., Wang R. (2018). Nanostructure Optimization of Platinum-Based Nanomaterials for Catalytic Applications. Nanomaterials.

[2] Antolini E., Lopes T., Gonzalez E.R. (2008). An overview of platinum-based catalysts as methanol-resistant oxygen reduction materials for direct methanol fuel cells. J. Alloys Compd..

[3] Hauch A., Georg A. (2001). Diffusion in the electrolyte and charge-transfer reaction at the platinum electrode in dye-sensitized solar cells. Electrochim. Acta.

[4] Giuseppe C., Pietro C., Alessia I., Alessandro S., Ilaria C., Gaetano D.M.A. (2011). new type of transparent and low cost counter-electrode based on platinum nanoparticles for dye-sensitized solar cells. Energy Environ. Sci..

[5] Yang M., Yang Y., Liu Y., Shen G., Yu R. (2006). Platinum nanoparticles-doped sol-gel/carbon nanotubes composite electrochemical sensors and biosensors. Biosens. Bioelectron..

[6] Pedone D., Moglianetti M., De Luca E., Bardi G., Pompa P.P. (2017). Platinum nanoparticles in nanobiomedicine. Chem. Soc. Rev..

[7] Rehman M.U., Yoshihisa Y., Miyamoto Y., Shimizu T. (2012). The anti-inflammatory effects of platinum nanoparticles on the lipopolysaccharide-induced inflammatory response in RAW 264.7 macrophages. Inflamm. Res..

[8] Kim W., Lee J.S., Shin D.H., Jang J. (2018). Platinum nanoparticles immobilized on polypyrrole nanofibers for non-enzyme oxalic acid sensor. J. Mater. Chem. B.

[9] Suresh A., Novak S., Wellenius P., Misra V., Muth V.F. (2009). Transparent indium gallium zinc oxide transistor based floating gate memory with platinum nanoparticles in the gate dielectric. Appl. Phys. Lett..

[10] Chen A., Holt-Hindle P. (2010). Platinum-Based Nanostructured Materials: Synthesis, Properties, and Applications. Chem. Rev..

[11] Peng Z., Yang H. (2009). Designer platinum nanoparticles: Control of shape, composition in alloy, nanostructure and electrocatalytic property. Nano Today.

[12] Stepanov A.L., Golubev A.N., Nikitin S.I., Osin Y.N. (2014). A review on the fabrication and properties of platinum nanoparticles. Rev. Adv. Mater. Sci..

[13] San B.H., Moh S.H., Kim K.K. (2013). Investigation of the heating properties of platinum nanoparticles under a radiofrequency current. Int. J. Hyperthermia.

[14] Johannsen M., Thiesen B., Wust P., Jordan A. (2010). Magnetic nanoparticle hyperthermia for prostate cancer. Int. J. Hyperthermia.

[15] Brondani D., Scheeren C.W., Dupont J., Vieira I.C. (2009). Biosensor based on platinum nanoparticles dispersed in ionic liquid and laccase for determination of adrenaline. Sens. Actuators B Chem..

[16] Ullah M.H., Chung W.S., Kim I., Ha C.S. (2006). pH-selective synthesis of monodisperse nanoparticles and 3D dendritic nanoclusters of CTAB-stabilized platinum for electrocatalytic O_2_ reduction. Small.

[17] Du Y.K., Yang P., Mou Z.G., Hua N., Jiang L. (2006). Thermal decomposition behaviors of PVP coated on platinum nanoparticles. J. Appl. Polym. Sci..

[18] Wang X., Zhang Y., Li T., Tian W., Zhang Q., Cheng Y. (2013). Generation 9 Polyamidoamine Dendrimer Encapsulated Platinum Nanoparticle Mimics Catalase Size, Shape, and Catalytic Activity. Langmuir.

[19] Schmidt E., Kleist W., Krumeich F., Mallat T., Baiker A. (2010). Platinum Nanoparticles: The Crucial Role of Crystal Face and Colloid Stabilizer in the Diastereoselective Hydrogenation of Cinchonidine. Chem. Eur. J..

[20] Fu Y., Zhao X., Zhang J., Li W. (2014). DNA-Based Platinum Nanozymes for Peroxidase Mimetics. J. Phys. Chem. C.

[21] Yu C.J., Chen T.H., Jiang J.Y., Tseng W.L. (2014). Lysozyme-directed synthesis of platinum nanoclusters as a mimic oxidase. Nanoscale.

[22] He S.B., Deng H.H., Liu A.L., Li G.W., Lin X.H., Chen W., Xia X.H. (2014). Synthesis and Peroxidase-Like Activity of Salt-Resistant Platinum Nanoparticles by Using Bovine Serum Albumin as the Scaffold. Chem. Cat. Chem..

[23] Chen X., Wu G., Chen J., Chen X., Xie Z., Wang X. (2011). Synthesis of “clean” and well-dispersive Pd nanoparticles with excellent electrocatalytic property on graphene oxide. J. Am. Chem. Soc..

[24] Das R., Nath S.S., Chakdar D., Gope G., Bhattacharjee R. (2010). Synthesis of silver nanoparticles and their optical properties. J. Exp. Nanosci..

[25] Gharibshahi E., Saion E., Ashraf A., Gharibshahi L. (2017). Size-Controlled and Optical Properties of Platinum Nanoparticles by Gamma Radiolytic Synthesis. Appl. Radiat. Isot..

[26] Mendivil Palma M.I., Krishnan B., Rodriguez G.A.C., Das Roy T.K., Avellaneda D.A., Shaji S. (2016). Synthesis and Properties of Platinum Nanoparticles by Pulsed Laser Ablation in Liquid. J. Nanomater..

[27] Jakobi J., Menéndez-Manjón A., Chakravadhanula V.S., Kienle L., Wagener P., Barcikowski S. (2011). Stoichiometry of alloy nanoparticles from laser ablation of PtIr in acetone and their electrophoretic deposition on PtIr electrodes. Nanotechnology.

[28] Yan Z., Bao R., Chrisey D.B. (2010). Excimer laser ablation of a Pt target in water: The observation of hollow particles. Nanotechnology.

[29] Mendivil M., Krishnan B., Castillo G.A., Shaji S. (2015). Synthesis and properties of palladium nanoparticles by pulsed laser ablation in liquid. Appl. Surf. Sci..

[30] Goncalves G., Marques P.A.A.P., Granadeiro C.M., Nogueira H.I.S., Singh M.K., Grácio J. (2009). Surface Modification of Graphene Nanosheets with Gold Nanoparticles: The Role of Oxygen Moieties at Graphene Surface on Gold Nucleation and Growth. Chem. Mater..

[31] Chen C., Long M.C., Wu H.D., Cai W.M. (2013). One-step synthesis of Pt nanoparticles/reduced graphene oxide composite with enhanced electrochemical catalytic activity. Sci. China Chem..

[32] Sadrolhosseini A.R., Noor A.S.M., Shameli K., Kharazmi A., Huang N.M., Mahdi M.A. (2013). Preparation of graphene oxide stabilized nickel nanoparticles with thermal effusivity properties by laser ablation method. J. Nanomater..

[33] Hontoria-Lucas C., Lopez-Peinado A.J., Lopez-Gonzalez J.d.D., Rojas-Cervantes M.L., Martin-Aranda R.M. (1995). Study of oxygen-containing groups in a series of graphite oxides: Physical and chemical characterization. Carbon.

[34] Kim W.J., Basavaraja C., Thinh P.X., Huh D.S. (2013). Structural characterization and DC conductivity of honeycomb-patterned poly(ε-caprolactone)/gold nanoparticle-reduced graphite oxide composite films. Mater. Lett..

[35] Emiru T.F., Ayele D.W. (2017). Controlled synthesis, characterization and reduction of graphene oxide: A convenient method for large scale production. Egypt. J. Basic Appl. Sci..

[36] Ge S., Yan M., Lu J., Zhang M., Yu F., Yu J., Song X., Yu S. (2012). Electrochemical biosensor based on graphene oxide–Au nanoclusters composites for l-cysteine analysis. Biosens. Bioelectron..

[37] Ren P.G., Yan D.X., Ji X., Chen T., Li Z.M. (2010). Temperature dependence of graphene oxide reduced by hydrazine hydrate. Nanotechnology.

[38] Niu Z., Chen J., Hng H.H., Ma J., Chen X. (2012). A leavening strategy to prepare reduced graphene oxide foams. Adv. Mater..

[39] Sadrolhosseini A.R., Noor A.S.M., Faraji N., Kharazmi A., Mahdi M.A. (2014). Optical Nonlinear Refractive Index of Laser-Ablated Gold Nanoparticles Graphene Oxide Composite. J. Nanomater..

[40] Fu Q., Sun W. (2001). Mie theory for light scattering by a spherical particle in an absorbing medium. Appl. Opt..

[41] Walter J.G., Petersen S., Stahl F., Scheper T., Barcikowski S. (2010). Laser ablation-based one-step generation and bio-functionalization of gold nanoparticles conjugated with aptamers. J. Nanobiotechnol..

[42] Gupta P., Ramrakhiani M. (2009). Influence of the Particle Size on the Optical Properties of CdSe Nanoparticles. Open Nanosci. J..

[43] Sadrolhosseini A.R., Rashid S.A., Shafie S., Soleimani H. (2019). Laser ablation synthesis of Ag nanoparticles in graphene quantum dots aqueous solution and optical properties of nanocomposite. Appl. Phys. A.

[44] Zamiri R., Zakaria A., Ahangar H.A., Sadrolhosseini A.R., Mahdi M.A. (2010). Fabrication of silver nanoparticles dispersed in palm oil using laser ablation. Int. J. Mol. Sci..

[45] Fox M. (2002). Optical Properties of Solids.

[46] Rad A.G., Abbasi H., Golyari K. (2012). Fabrication and nonlinear refractive index measurement of colloidal silver nanoparticles. Int. J. Appl. Phys. Math..

[47] Sheik-Bahae M., Said A.A., Van Stryland E.W. (1989). High-sensitivity, single-beam n 2 measurements. Opt. Lett..

[48] Prusty S., Mavi H.S., Shukla A.K. (2005). Optical nonlinearity in silicon nanoparticles: Effect of size and probing intensity. Phys. Rev. B.

[49] Sadrolhosseini A.R., Noor A.S.M., Mehdipour L.A., Noura A., Mahdi M.A. (2015). Application of thermal lens technique to measure the thermal diffusivity of biodiesel blend. Opt. Rev..

[50] Shen J., Lowe R.D., Snook R.D. (1992). A model for cw laser induced mode-mismatched dual-beam thermal lens spectrometry. Chem. Phys..

[51] Delgado-Vasallo O., Marin E. (1999). Application of the photoacoustic technique to the measurement of the thermal effusivity of liquids. J. Phys. D.

[52] Sadrolhosseini A.R., Rashid S.A., Noor A.S.M., Kharazmi A., Mehdipour L.A. (2015). Fabrication of silver nanoparticles in pomegranate seed oil with thermal properties by laser ablation technique. Dig. J. Nanomater. Biostruct..

[53] Huang N.M., Lim H.N., Chia C.H., Yarmo M.A., Muhamad M.R. (2011). Simple room-temperature preparation of high-yield large-area graphene oxide. Int. J. Nanomed..

[54] Sadrolhosseini A.R., Mahdi M.A., Alizadeh F., Rashid S.A. (2018). Laser Ablation Technique for Synthesis of Metal Nanoparticle in Liquid. Laser Technology and its Applications.

[55] Zhang J., Claverie J., Chaker M., Ma D. (2017). Colloidal Metal Nanoparticles Prepared by Laser Ablation and their Applications. ChemPhysChem.

[56] Ratti M., Naddeo J.J., Griepenburg J.C., O’Malley S.M., Bubb D.M., Klein E.A. (2017). Production of Metal Nanoparticles by Pulsed Laser-ablation in Liquids: A Tool for Studying the Antibacterial Properties of Nanoparticles. J. Vis. Exp..

[57] Zhang Y., Wang Y. (2017). Nonlinear optical properties of metal nanoparticles: A review. RSC Adv..

[58] Sadrolhosseini A.R., Abdul Rashid S., Shojanazeri H., Noor A.S.M. (2016). Spatial self-phase modulation patterns in graphene oxide and graphene oxide with silver and gold nanoparticles. Opt. Quant. Electron..

[59] Deng L., He K., Zhou T., Li C. (2005). Formation and evolution of far-field diffraction patterns of divergent and convergent Gaussian beams passing through self-focusing and self-defocusing media. J. Opt. A Pure Appl. Opt..

[60] Zamiri R., Parvizi R., Zakaria A., Sadrolhosseini A.R., Zamiri G., Darroudi M., Husin M.S. (2012). Investigation on nonlinear-optical properties of palm oil/silver nanoparticles. J. Eur. Opt. Soc. Rapid Publ..

[61] Karimzadeh K. (2012). Spatial self-phase modulation of a laser beam propagating through liquids with self-induced natural convection flow. J. Opt..

[62] Zamiri R., Azmi Z., Bin Ahmad M., Shameli K., Darroudi M., Mahdi M.A., Husin M.S. (2010). Thermal diffusivity of silver metallic nanoparticles in clay matrix. J. Optoelectron. Adv. Mater..

[63] Snook R.D., Lowe R.D. (1995). Thermal lens spectrometry A review. Analyst.

[64] Silva R., de Araújo M.A., Jali P., Moreira S.G., Alcantara P., de Oliveira P.C. (2011). Thermal lens spectrometry: Optimizing amplitude and shortening the transient time. AIP Adv..

[65] Gao F., Feng X., Zhang R., Liu S., Ding R., Kishor R., Zheng Y. (2017). Single laser pulse generates dual photoacoustic signals for differential contrast photoacoustic imaging. Sci. Rep..

